# A Novel Genetic Engineering Approach for DON Detoxification Using a Yeast-Based Multi-Enzyme System

**DOI:** 10.3390/biology15080654

**Published:** 2026-04-21

**Authors:** Rong Li, Jia Song, Bo Sun, Aike Li, Shiqi Zou, Ming Liu, Linshu Jiang, Jingjing Shi, Qingming Cao, Chen Zhao, Weiwei Wang

**Affiliations:** 1 Animal Science and Technology College, Beijing University of Agriculture, Beijing 102206, China; lirong20221018@163.com (R.L.); liuming@bua.edu.cn (M.L.); jls@bua.edu.cn (L.J.); 2 Key Laboratory of Grain and Oil Biotechnology, Academy of National Food and Strategic Reserves Administration, Beijing 100037, China; songj@ags.ac.cn (J.S.); sunb@ags.ac.cn (B.S.); lak@ags.ac.cn (A.L.); 19375100386@163.com (S.Z.); sjj@ags.ac.cn (J.S.); 3 College of Food Science and Engineering, Central South University of Forestry & Technology, Changsha 410004, China; cqm2000cn@163.com

**Keywords:** genetically engineered yeast, pyrroloquinoline quinone (PQQ), toxin detoxification, *Saccharomyces cerevisiae*

## Abstract

Deoxynivalenol (DON), a Group III carcinogenic mycotoxin frequently detected in cereals and animal-derived food products, poses serious health risks to animals and humans. In this study, we developed a genetically engineered *Saccharomyces cerevisiae* strain as a proof-of-concept platform for DON detoxification. The yeast was engineered to co-express two detoxification genes, *YTDepA* and *YTDepB* (homologs of *DepA* and *DepB* from *Devosia mutans* 17-2-E-8) originally identified in *Youhaiella tibetensis*. Concurrently, the pyrroloquinoline quinone (PQQ) biosynthesis gene cluster from *Klebsiella pneumoniae* was integrated to supply the essential cofactor. Gene expression was verified by qRT-PCR and Western blot. The recombinant strain demonstrated a significant 13.98% detoxification of DON after 72 h of fermentation (*p* < 0.05), as confirmed by HPLC–MS, while the strain expressing only the PQQ cluster showed no detoxification activity. This study establishes an integrated yeast cell factory for DON detoxification and highlights key limitations to guide future optimization efforts.

## Simple Summary

Grains and cereals are frequently contaminated by a harmful mold toxin deoxynivalenol (DON), which can cause serious health issues for both humans and livestock. Since this toxin is difficult to be removed during regular food and feed processing, our study aimed to create a safe and natural solution using common baker’s yeast. We genetically modified the yeast to act as a tiny “cleanup crew” by giving it the biological tools needed to break down the toxin into harmless components. To ensure these tools worked correctly, we also engineered the yeast to produce its own internal supply of a dedicated molecule pyrroloquinoline quinone (PQQ) required for the reaction. Our results showed that this modified yeast successfully neutralized nearly 14% of DON within three days of cultivation using the engineered yesat. While this is an initial step, the study proves that we can turn simple microorganisms into specialized factories to detoxify our food supply. This research provides a vital foundation for developing more efficient ways to protect global food safety and reduce the waste caused by mold-damaged crops.

## 1. Introduction

Deoxynivalenol (DON), a mycotoxin produced by *Fusarium* species, is a pervasive contaminant in cereals and their derivatives. Due to its stability under high temperatures and acidic conditions, DON resists conventional detoxification methods [1], making it a significant threat to both human and animal health. It is classified as a Group III carcinogen and has been associated with various health risks, including immune suppression, oxidative damage, and reproductive dysfunction [1,2]. Beyond its toxic effects, DON also causes substantial economic losses globally [3]. The key toxic moieties of DON, the C-12,13 epoxy group and the C3-OH group (Figure S1) [4], are difficult to detoxify through traditional physical or chemical means, such as thermal treatment, adsorption, or chemical oxidation, primarily because DON is highly stable. Furthermore, these methods often cause a reduction in the nutritional value of food and feed, or generate undesirable toxic by-products [1]. Therefore, biological detoxification offers a promising alternative for managing DON contamination by employing microorganisms or enzymes to convert DON into less toxic derivatives [5].

A two-step enzymatic process has been reported for *Devosia mutans* 17-2-E-8 (Figure S2), where a pyrroloquinoline quinone (PQQ)-dependent dehydrogenase (DepA) oxidizes DON to 3-keto-DON, followed by NADPH-dependent reduction (DepB) to 3-epi-DON [6,7]. Notably, the *Devosia* strain D6–9 utilizes a similar mechanism involving a PQQ-dependent dehydrogenase QDDH and NADPH-dependent reductases AKR13B2/AKR6D1 for subsequent reduction to 3-epi-DON [8,9]. A similar enzymatic strategy occurs in *Youhaiella tibetensis* F4T (recently reclassified in January 2025 and also deposited in databases under the homotypic synonym *Paradevosia tibetensis* [10]), a strain isolated from permafrost, which harbors homologs of these enzymes (YTDepA and YTDepB) exhibiting high sequence similarity (93.3% and 88.0%, respectively) to their counterparts in *D. mutans* [11]. Recent studies have confirmed that YTDepA (YoDDH) exhibits superior kinetic parameters for DON dehydrogenation [4]. In this pathway, PQQ plays a crucial role as a redox cofactor in alcohol dehydrogenases, facilitating electron transfer and alcohol oxidation to aldehydes or ketones via Ca^2+^-binding domains [12]. PQQ is naturally synthesized by *Klebsiella pneumonia* through a six-gene cluster (*pqqABCDEF*) [13], with the biosynthesis initiated by the precursor peptide PqqA [14]. The subsequent enzymes, including PqqD [15], PqqE [15], PqqF [16], PqqB [17], and PqqC, catalyze a series of reactions to complete the PQQ formation [15].

Historically, biological approaches have predominantly relied on either the direct application of native DON-detoxifying bacteria or the *in vitro* use of purified recombinant enzymes. However, both past methods present significant practical limitations. First, native DON-detoxifying strains, such as those from the *Devosia* genus, are typically environmental isolates that lack the Generally Recognized As Safe (GRAS) status, strictly limiting their direct application in the food and feed industries. Second, utilizing purified enzymes *in vitro* (such as the DepA/DepB system) necessitates the external supplementation of expensive cofactors, particularly PQQ and NADPH, which dramatically increases operational costs and restricts large-scale industrial viability.

To overcome these limitations, our current approach utilizes *Saccharomyces cerevisiae*, a well-established GRAS microorganism widely used in food processing, as a host. While the two-step enzymatic epimerization of DON has been reported [6,7], the construction of a unified, self-sufficient microbial cell factory that harbors both the complete detoxification pathway (YTDepA & YTDepB) and an endogenous cofactor supply system (PQQ biosynthesis cluster) within a single yeast host remains an underexplored yet promising strategy. This integrated system fundamentally eliminates the need for expensive external cofactor supplementation and bypasses the safety concerns of using non-GRAS microorganisms. Therefore, this study aims to design, construct, and preliminarily evaluate a genetically engineered *S. cerevisiae* strain capable of concurrently producing the DON-detoxifying enzymes and their required cofactor. The successful implementation of this integrated approach is expected to establish a foundational platform for the development of efficient and economical biocatalysts for DON detoxification in industrial applications.

## 2. Materials and Methods

### 2.1. Strains and Plasmids

Key strains and plasmids constructed and used in this study are listed in Table 1. Information on other plasmids is provided in Table S1. Simplified designations are used throughout the text for clarity.

### 2.2. Gene Cloning, Expression, and Functional Characterization of DON Oxidase

A gene encoding a DON oxidase, showing 93% sequence similarity to *DepA* (accession no. KFL25551.1) from *D. mutans* 17-2-E-8, was identified in *Y. tibetensis* through sequence alignment and designated *YTDepA* (accession no. GGF34699.1). As previously reported, DepA requires PQQ and Ca^2+^ as essential cofactors for the detoxification of DON [6]. Therefore, PQQ and Ca^2+^ were supplemented in all reaction systems. A mutant DON oxidase, TDDH, with reported higher *Vmax* than DepA [18], was synthesized using the plasmid pUC57-TDDH and amplified for further studies.

To ensure optimal protein expression in the yeast host, the nucleotide sequences of the target genes (*YTDepA*, *TDDH*, *YTDepB*, and the *pqq* cluster) were codon-optimized based on the codon bias of *S. cerevisiae* and chemically synthesized by Sangon Biotech (Shanghai, China). The primer sequences involved are provided in Table S2. The genes *YTDepA* and *TDDH* were, respectively, cloned into the expression vectors pETDuet and pET24a, resulting in the recombinant constructs pETDuet-YTDepA and pET24a-TDDH. These plasmids were transformed into *E. coli* BL21 (DE3) for recombinant protein expression. The target proteins were purified using affinity chromatography and incubated with DON in the presence of PQQ and Ca^2+^ to assess their detoxification efficiency and identify reaction products. The optimal pH and metal ion conditions for YTDepA activity were determined by measuring detoxification rates under various conditions.

### 2.3. Optimization of YTDepA Reaction Conditions

All reactions were performed in triplicate with a total volume of 1 mL. The following conditions were optimized:

Metal Ion Specificity: Purified YTDepA (15 μg) was incubated with 100 μM PQQ, 50 μg/mL DON in 50 mM Tris-HCl (pH 7.5), and 1 mM of various metal ions (Ca^2+^, Co^2+^, Cu^2+^, Fe^3+^, K^+^, Mg^2+^, Mn^2+^, Zn^2+^). A control without metal ions was included. Reactions were carried out at 30 °C with shaking for 24 h.

Optimal pH: The pH optimum for YTDepA activity was determined using different buffers: acetic acid buffer (pH 3.0–6.0), phosphate buffer (pH 6.0–7.5), and Tris-HCl buffer (pH 7.5–9.0). Reactions were conducted in the presence of 15 μg YTDepA, 1 mM Ca^2+^, 100 μM PQQ, and 50 μg/mL DON. Controls without enzyme were also included. Reactions were incubated at 30 °C for 24 h.

### 2.4. Expression, Refolding and Activity Assay of DON Reductase YTDepB

A gene encoding a DON reductase, *YTDepB*, with 88% similarity to *DepB* (accession no. KFL28068.1) from *D. mutans*, was identified in *Y. tibetensis* (accession no. WP_188569619.1). Since NADPH has been shown to significantly enhance the production of 3-epi-DON by DepB by 15-fold, YTDepB was also assumed to be NADPH-dependent [19]. The *YTDepB* gene was cloned into pET24a and expressed in *E. coli* BL21 (DE3), yielding the recombinant strain pET24a-YTDepB-BL21.

SDS-PAGE analysis confirmed that YTDepB was expressed predominantly as insoluble inclusion bodies. Soluble protein was recovered by denaturation and refolding methods [20,21,22]. The enzymatic activity of YTDepB was verified by measuring DON detoxification in the presence of NADPH and 3-keto-DON.

### 2.5. Biosynthesis and Detection of Cofactor PQQ in Recombinant Yeast

*S. cerevisiae* CEN.PK2 was used as the host strain for cofactor PQQ production. The PQQ synthesis gene clusters (*PqqA*, *PqqB*, *PqqC*, *PqqF*) were integrated into the YPRCΔ15 locus on chromosome XVI, while *PqqD* and *PqqE* were integrated into the YORWΔ17 locus on chromosome XV [23]. The recombinant constructs pESCtp01-KppqqAF, pESCtp01-KPpqqBC, and pESCtp01-KppqqDE were generated and inserted into the pUC57 vector containing the respective chromosomal integration sites. Two plasmids, pUC57-Y15site1-KPpqqAF-BC-Y15site2 and pUC57-Y17site1-KPpqqDE-Y17site2, were obtained and digested for linearization. 5 μg of the linearized pUC57-Y15site1-KPpqqAF-BC-Y15site2 plasmid was added to 200 μL of CEN.PK2 competent cells in a 0.2 cm electroporation cuvette, and electroporation was performed at 2000 V, 25 μF, and 200 Ω. Following electroporation, the cells were plated on SC-TRP selection medium (SC medium lacking tryptophan) for cultivation. A single white colony of pUC57-Y15site1-KppqqAF-BC-Y15site2-CEN.PK2 grown on the plate was selected and used to prepare competent cells. Five micrograms of the linearized pUC57-Y17site1-KPpqqDE-Y17site2 plasmid was then added to 200 μL of these competent cells in a 0.2 cm electroporation cuvette. After electroporation, the cells were plated on SC-TRP-LEU selection medium (SC medium lacking tryptophan and leucine) and incubated at 30 °C for 3–5 days. The resulting white colonies, designated *Sc*-PQQ, represented the complete construct for PQQ production in *S. cerevisiae*.

PQQ production was monitored by high-performance liquid chromatography ThermoFisher UltiMate 3000 (HPLC) (Thermo Fisher Scientific, Waltham, MA, USA) using a COSMOSIL 5C18-MS-II column (4.6 × 150 mm, 5 μm) (Nacalai Tesque, Kyoto, Japan) at 25 °C. The mobile phase consisted of methanol (A) and water (adjusted to pH 1.0 with trifluoroacetic acid) (B), and elution was carried out under a gradient. The gradient elution program was as follows: 0–0.40 min, 20% B; 0.40–1.00 min, 20–70% B; 1.00–2.50 min, 70% B; 2.50–2.51 min, 70–20% B; 2.51–3.00 min, 20% B. The total run time was 3 min, the flow rate was 0.3 mL/min, and the injection volume was 2 µL. For LC-MS/MS (Agilent UPLC 1290-qTOF 6545, Agilent Technologies, Santa Clara, CA, USA) analysis, a Thermo UHPLC column (Thermo Fisher Scientific, Waltham, MA, USA) was employed with a mobile phase of ammonium acetate and acetonitrile. The PQQ content was detected using electrospray ionization in negative mode (ESI-). The PQQ concentration was quantified by LC-MS using the supernatant of fermentation, in which an equal volume of methanol was added for sample preparation.

### 2.6. Co-Expression of DON Detoxification Enzymes and PQQ in Yeast

The genes encoding YTDepA and YTDepB were amplified and fused to the α-factor signal peptide sequence from plasmid pPinkα-HC to direct the recombinant proteins through the endoplasmic reticulum (ER) for extracellular secretion. This design ensures that the enzymes can be secreted into the extracellular environment to directly interface with the target mycotoxin. The constructs pESCtp01-TEF-α-YTDepA and pESCtp01-TEF-α-YTDepA-PGK1-α-YTDepB were sequentially generated using pESCtp01 as the backbone. These expression cassettes were integrated into the vector pUC57-Ty1-2, containing a Ty transposon site for genomic integration in *S. cerevisiae*, resulting in pUC57-Ty1-2-TEF1-α-YTDepA-PGK1-α-YTDepB. This construct was transformed into the recombinant *S. cerevisiae* strain producing PQQ (from Section 2.4). Integration and expression were confirmed by qRT-PCR and Western blot. The recombinant yeast strain was assessed for its ability to detoxify DON.

### 2.7. Analytical Methods for DON and Metabolite Quantification

DON concentration and its detoxification products were quantified using high-performance liquid chromatography (HPLC) and liquid chromatography–mass spectrometry (LC-MS). Chromatographic separations were performed using an ultra-high-pressure column (100 × 2.1 mm) (Thermo Fisher Scientific, San Jose, CA, USA) maintained at 30 °C, with a mobile phase of methanol:water (20:80, *v*/*v*) delivered at 0.3 mL/min. The injection volume was 2 μL. For structural identification and confirmatory quantification, LC-MS was employed using an electrospray ionization source in positive mode (ESI+). A standard curve was constructed using DON solutions in deionized water at concentrations of 0.5, 1, 5, 10, 50, and 100 μg/mL. Samples were analyzed in triplicate.

#### 2.7.1. DON Detoxification by Recombinant YTDepA and Efficiency Assessment

The detoxification of DON by YTDepA was assessed by incubating purified enzyme (5 μg) with 50 μg/mL DON in 40 mM Tris buffer (pH 7.5). After incubation at 30 °C with shaking, aliquots were taken at 0, 2, 4, 8, and 12 h. The reactions were terminated by adding an equal volume of methanol, followed by centrifugation at 12,000 rpm for 10 min. The detoxification efficiency was calculated based on the residual DON concentration determined by HPLC. The major detoxification product was identified as 3-keto-DON by LC-MS via comparison of its retention time and mass spectrum with an authentic standard.

#### 2.7.2. Detoxification of 3-Keto-DON by Recombinant YTDepB and Product Identification

The activity of YTDepB toward 3-keto-DON was evaluated by incubating 5 μg of refolded YTDepB with 5 μg/mL 3-keto-DON and 2 mM NADPH in 40 mM Tris buffer (pH 7.5). Control reactions received an equivalent amount of heat-inactivated enzyme. Reactions were performed in triplicate at 30 °C with shaking, and aliquots (100 μL) were collected at 0, 12, 24, and 48 h and processed as described in Section 2.2. The substrate 3-keto-DON was monitored and confirmed by LC-MS under the same ESI+ parameters described in Section 2.1, based on its characteristic ion spectrum and a distinct retention time shift.

#### 2.7.3. Detoxification Efficiency and Metabolic Profile of the Engineered Yeast Strain

The engineered *S. cerevisiae* strain expressing YTDepA, YTDepB, and PQQ was tested for its ability to detoxify DON in a complete biotransformation pathway. As a control, the strain harboring only the PQQ biosynthesis genes (*Sc*-PQQ) was incubated under identical conditions to account for any non-specific loss of DON. The strain was inoculated (2%, *v*/*v*) into 40 mL of YPD medium containing 1 μg/mL DON. Triplicate cultures were incubated at 30 °C with shaking at 200 rpm. Samples (1 mL) were collected at 0, 12, 24, 48, and 72 h. The samples were centrifuged at 5000 rpm for 10 min, and the supernatant was recentrifuged at 10,000 rpm for 10 min to remove cells and debris. The final supernatant was filtered through a 0.45 μm membrane. The detoxification efficiency was determined by measuring the residual DON concentration via LC-MS. Metabolic intermediates, such as 3-keto-DON and 3-epi-DON, were monitored to assess the progress of the enzymatic isomerization.

### 2.8. Statistical Analysis

All experiments were conducted using a completely randomized design. The experimental unit was defined as an independent reaction tube (for *in vitro* enzymatic assays) or an independent shake flask culture (for yeast fermentation assays). All assays were performed with at least three independent biological replicates (*n* = 3), and data were presented as the mean ± standard deviation (SD). Statistical analysis was conducted using SAS 9.0 software (SAS Institute Inc., Cary, NC, USA). A one-way analysis of variance (ANOVA) statistical model was applied to evaluate the main effects of individual treatments (e.g., reaction time, metal ions, or pH) on DON detoxification. Differences among treatment means were determined using the Student–Newman–Keuls multiple-range test and were considered statistically significant at *p* < 0.05.

## 3. Results

### 3.1. Functional Characterization of Recombinant DON-Detoxifying Enzymes from Y. tibetensis

**Figure 1:**
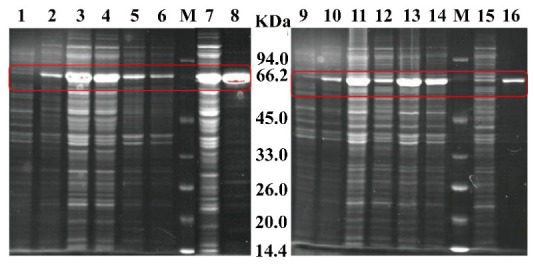
SDS-PAGE analysis of pETDuet-YTDepA-BL21 and pET24a-TDDH- BL21. The molecular masses of YTDepA and TDDH are approximately 61.9 kDa and 61.4 kDa, respectively. The left panel displays the SDS-PAGE analysis of pETDuet-YTDepA-BL21, the red square indicates the purified YTDepA band, while the right panel shows the SDS-PAGE analysis of pET24a-TDDH-BL21, the red square indicates the purified YTDepA band. In the figure, lanes are designated as follows: 1: YTDepA without inducer; 2: YTDepA with inducer; 3: total lysate of YTDepA; 4: supernatant of YTDepA lysate; 5: pelleted cells of YTDepA lysate concentrated 10-fold; 6: pelleted cells of YTDepA lysate concentrated 20-fold; 7: flow-through of YTDepA purification; 8: purified YTDepA fraction; 9: TDDH without inducer; 10: TDDH with inducer; 11: total lysate of TDDH; 12: supernatant of TDDH lysate; 13: pelleted cells of TDDH lysate concentrated 10-fold; 14: pelleted cells of TDDH lysate concentrated 20-fold; 15: flow-through of TDDH purification; 16: purified TDDH fraction; M: 14.4–94.0 kDa Protein Marker.

**Figure 2:**
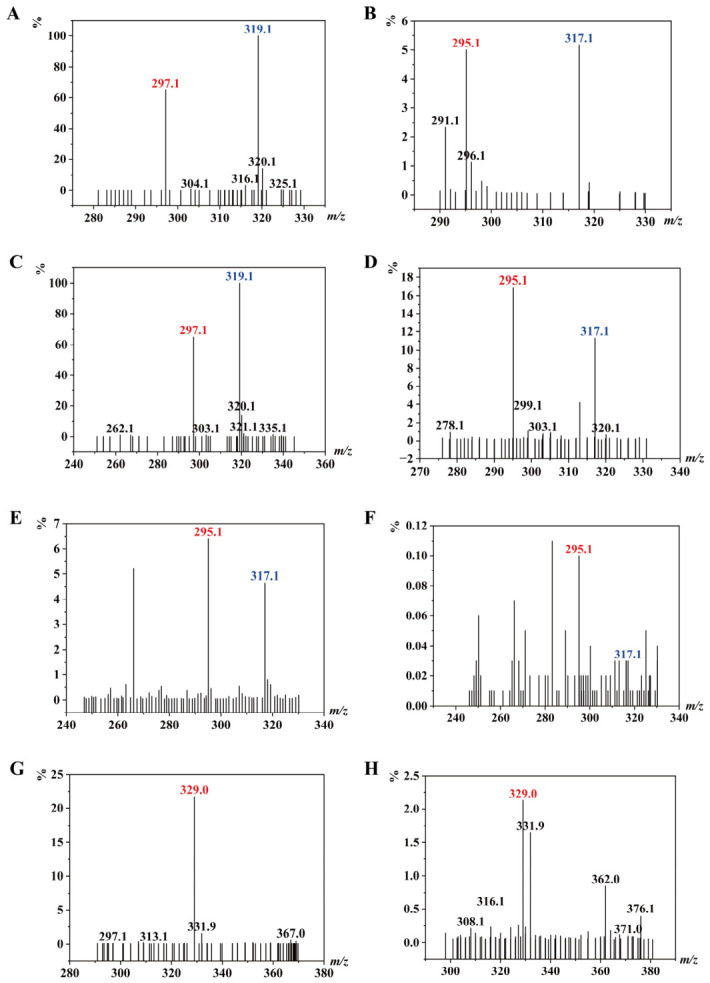
The red and blue labels represent the specific ion peaks of the detected compounds: *m*/*z* 297.1 (red) and 319.1 (blue) correspond to the [M + H]^+^ and [M + Na]^+^ peaks of DON, respectively; *m*/*z* 295.1 (red) and 317.1 (blue) correspond to the [M + H]^+^ and [M + Na]^+^ peaks of 3-keto-DON, respectively; *m*/*z* 329.0 (red) represents the [M − H]^−^ peak of PQQ. (**A**) Mass spectrum of DON at 0 h of reaction with YTDepA (positive ion mode). (**B**) Mass spectrum of DON at 48 h of reaction with YTDepA (positive ion mode). (**C**) Mass spectrum of DON at 0 h of reaction with TDDH (positive ion mode). (**D**) Mass spectrum of DON at 48 h of reaction with TDDH (positive ion mode). (**E**) Mass spectrum of 3-keto-DON at 0 h of reaction with YTDepB (positive ion mode). (**F**) Mass spectrum of 3-keto-DON at 48 h of reaction with YTDepB (positive ion mode). (**G**) Mass spectrum of the PQQ standard (negative ion mode). (**H**) Mass spectrum of the fermentation products from the recombinant strain (negative ion mode).

**Figure 3:**
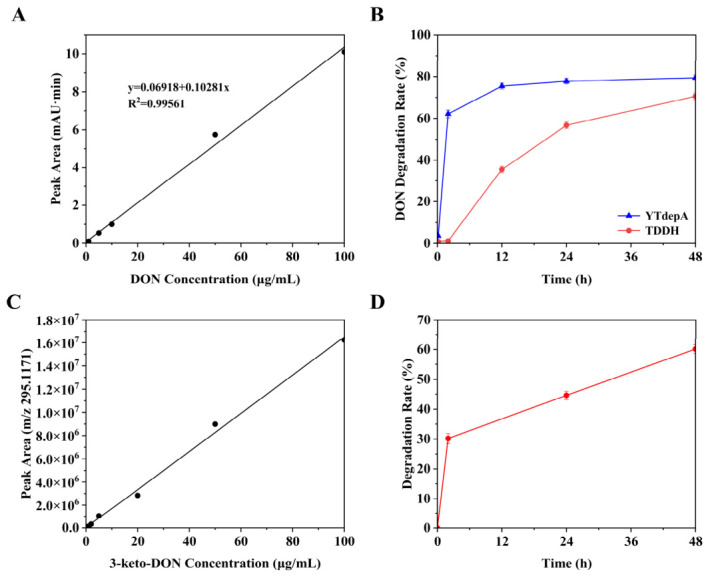
Standard curve and detoxification rate curves of different enzymes over time. (**A**) Standard curve of DON. (**B**) DON detoxification rates at different times of action of *YT*DepA and TDDH. (**C**) Standard curve of 3-keto-DON. (**D**) Detoxification rate of 3-keto-DON by YTDepB at different time points.

To harness the potential of *Y. tibetensis* for DON detoxification, we first cloned and expressed its two key enzymes, YTDepA and YTDepB, which share high sequence similarity with the well-characterized DepA (PQQ-dependent dehydrogenase) and DepB (NADPH-dependent reductase) from *D. mutans* [6,7]. Recombinant YTDepA and a reference mutant enzyme TDDH were successfully expressed in *E. coli* BL21 (DE3) and purified, as confirmed by SDS-PAGE (Figure 1). *In vitro* activity assays demonstrated that both purified YTDepA and TDDH could efficiently convert DON to 3-keto-DON in the presence of their respective cofactors (PQQ for YTDepA, NADPH for TDDH), as evidenced by the characteristic mass shift from *m*/*z* 297 to 295 in LC-MS analysis (Figure 2A–D). A time-course experiment (Figure 3B) revealed that YTDepA exhibited a rapid initial reaction rate, achieving a final DON detoxification of 79.46% after 48 h, outperforming TDDH (70.58%). These results confirm that YTDepA is a highly active PQQ-dependent dehydrogenase suitable for further engineering.

**Figure 4:**
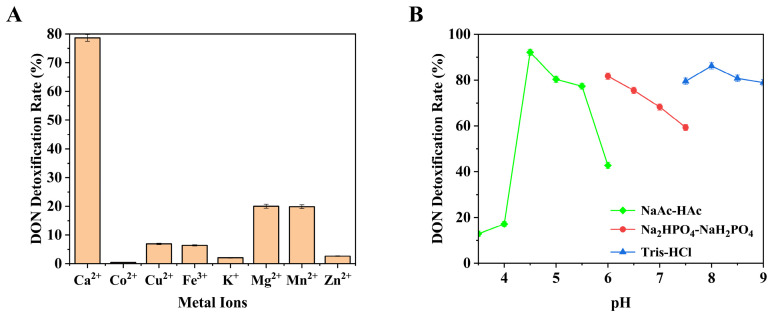
Effect of metal ions (**A**) and pH (**B**) on the detoxification rate of DON.

**Figure 5:**
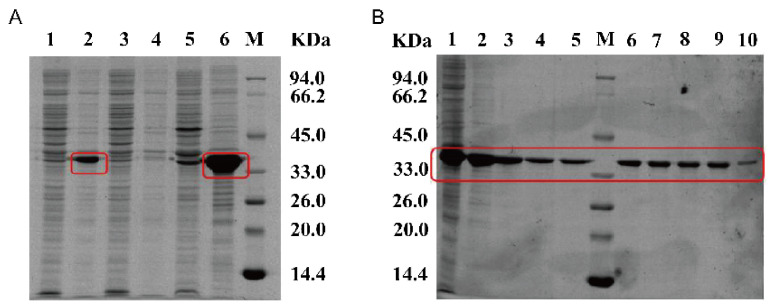
SDS-PAGE analysis of pET24a-YTDepB-BL21 (**A**) and its protein inclusion body refolding (**B**). (**A**) Lane 1: uninduced total lysate; 2: induced total lysate; 3: uninduced supernatant; 4: induced supernatant.; 5: uninduced pellet; 6: induced pellet; M: 14.4–94.0 kDa Protein Marker. The molecular mass of YTDepB is approximately 34.9 kDa. The red square indicates the purified YTDepB band. (**B**) Lane 1: PBS suspension; 2: wash suspension a.; 3: wash suspension b; 4: PBS (8 M Urea) solubilization fraction; 5: sample at 0 h of dialysis; 6: sample at 6 h of dialysis; 7: sample at 12 h of dialysis; 8: sample at 18 h of dialysis; 9: sample at 24 h of dialysis; 10: sample at 30 h of dialysis; M: 14.4–94.0 kDa Protein Marker. The red square indicates the YTDepB band after inclusion body refolding.

To optimize YTDepA activity, we systematically evaluated the effects of metal ions and pH. Ca^2+^ was identified as the most critical activator, while Mg^2+^ and Mn^2+^ provided moderate support (Figure 4A). The enzyme showed a distinct pH preference, with optimal activity observed at pH 4.5 in acetate buffer (Figure 4B). This pronounced dependence on Ca^2+^ and an acidic environment aligns with the known biochemistry of PQQ-dependent alcohol dehydrogenases [12] and provides crucial guidance for subsequent application in microbial hosts. Separately, the reductase YTDepB was expressed in *E. coli*, albeit primarily in the form of inclusion bodies (Figure 5A). Following denaturation and refolding (Figure 5B), the solubilized protein was confirmed to be active. When incubated with the product of the first step, 3-keto-DON, and NADPH, refolded YTDepB catalyzed its conversion, with a 48-h detoxification efficiency of 60.12% (Figure 3D) and the likely formation of 3-epi-DON (Figure 2E,F). This verifies that the *Y. tibetensis*-derived YTDepA/YTDepB pair constitutes a complete, functional two-step enzymatic module for DON epimerization *in vitro*.

### 3.2. Engineering S. cerevisiae for De Novo Biosynthesis of the Cofactor PQQ

A critical hurdle for deploying the PQQ-dependent YTDepA in a heterologous host is the supply of the cofactor. To address this, we engineered *S. cerevisiae* CEN.PK2 to biosynthesize PQQ *de novo*. The entire PQQ biosynthesis gene cluster (*pqqABCDEF*) from *K. pneumoniae* was split and integrated into the yeast genome at designated chromosomal loci (construction details in Supplementary Figure S3). LC-MS analysis of the fermentation broth from the resulting strain (designated *Sc*-PQQ) detected a compound with the exact mass-to-charge ratio (*m*/*z* 329, [M − H]^−^) matching that of a pure PQQ standard (Figure 2G,H). Then, the concentration of PQQ was determined to be 8.54 μg/mL with the same method using the supernatant of fermentation at 24 h. This indicates successful heterologous expression and suggests the functional assembly of the PQQ biosynthetic pathway in yeast, fulfilling a prerequisite for constructing a self-sufficient biocatalyst.

### 3.3. Assembly and Performance of an Integrated Yeast Strain Co-Expressing the Complete Detoxification Pathway

**Figure 6:**
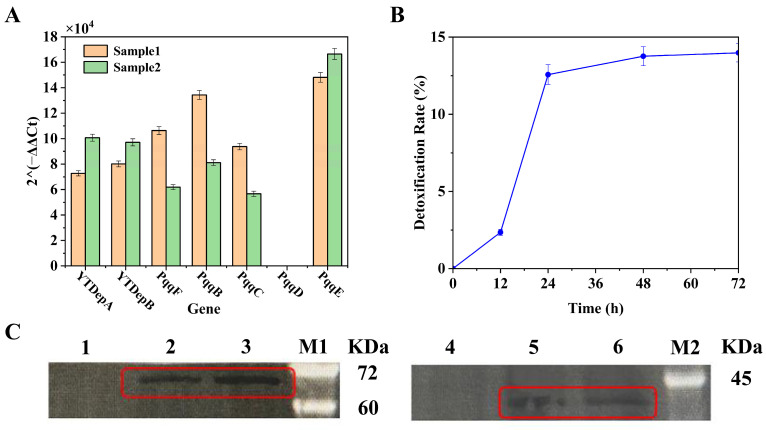
Verification of the engineered strain. (**A**) Relative expression levels of the target genes (2^-ΔΔCt^). Note: ΔCt = Ct (target gene)—Ct (internal reference gene). ΔΔCt = ΔCt (samples 1 & 2)—ΔCt (control CEN.PK2); (**B**) DON detoxification rate by the engineered strain co-expressing DON-detoxifying enzymes and PQQ over time; (**C**) Western blot analysis of target proteins YTDepA and YTDepB. In the left panel, the red square indicates the purified YTDepA band, with a molecular mass of 61.9 kDa. Lane 1: CEN.PK2 control; 2: YTDepA protein expression sample 1; 3: YTDepA protein expression sample 2. In the right panel, the red square indicates the purified YTDepB band, with a molecular mass of 34.9 kDa. Lane 4: CEN.PK2 control; 5: YTDepB protein expression sample 1; 6: YTDepB protein expression sample 2; M1 and M2: Protein Marker.

With the functional enzyme pair and the cofactor production module in hand, we aimed to integrate them into a single yeast cell. The genes encoding YTDepA and YTDepB, fused to secretion signal peptides, were integrated into the genome of the PQQ-producing strain (*Sc*-PQQ), yielding the final engineered strain *Sc*-PQQ-Dep. Molecular validation by qRT-PCR and Western blot confirmed the successful expression of all heterologous components in *Sc*-PQQ-Dep (Figure 6A,C, original images of Figure 6C are shown in Figures S4 and S5). Notably, the transcriptional level of *PqqD*—a key chaperone in PQQ biosynthesis—was significantly lower than that of other *pqq* genes and the detoxification enzymes (Figure 6A), pinpointing a potential bottleneck in the cofactor supply chain. The core functionality of the integrated system was then tested. When cultured in the presence of DON, strain *Sc*-PQQ-Dep achieved a 13.98% detoxification after 72 h (Figure 6B). Crucially, the control strain *Sc*-PQQ (producing only PQQ but lacking YTDepA/B) showed no significant DON detoxification under identical conditions, unequivocally attributing the detoxification activity to the introduced enzymatic pathway. This result provides direct *in vivo* evidence for the functional reconstitution of the complete DON-to-3-epi-DON conversion pathway within a recombinant yeast.

## 4. Discussion

Deoxynivalenol (DON) remains a formidable challenge in the agricultural and food sectors due to its potent ability to inhibit eukaryotic protein synthesis by targeting the 60S ribosomal subunit [24]. Developing biologically safe and self-sufficient detoxification platforms is therefore of great significance. Previous studies have extensively explored the use of *S. cerevisiae* for DON mitigation, but their mechanisms and outcomes differ fundamentally from our approach. Traditionally, wild-type *S. cerevisiae* reduces DON levels primarily through physical adsorption via its cell wall components (such as β-glucans and mannans). However, this physical binding is reversible and does not alter the toxic epoxide or hydroxyl groups, meaning the active toxin can easily be released again within the gastrointestinal tract [25]. Furthermore, earlier metabolic engineering efforts in yeast have sometimes focused on glycosylation (e.g., converting DON to DON-3-glucoside); yet, this merely generates a ‘masked mycotoxin’ that can be hydrolyzed back into the highly toxic DON by intestinal microbiota [26,27]. In contrast, the unique contribution of our research lies in achieving true, irreversible structural detoxification within a eukaryotic chassis. By introducing the complete DepA/DepB epimerization pathway, our engineered strain irreversibly converts DON into the essentially non-toxic 3-epi-DON. More importantly, by pioneering the *de novo* biosynthesis of the bacterial cofactor PQQ within the yeast chassis, we have created a truly self-sufficient whole-cell biocatalyst, successfully eliminating the prohibitive costs of external cofactor supplementation that hindered previous enzymatic approaches.

While the engineered strain *Sc*-PQQ-Dep in this study demonstrates a clear proof-of-concept, its current detoxification efficiency is lower than that observed with the purified enzymes *in vitro* or their native donor microorganisms. For context, the wild-type gene donor *D. mutans* 17-2-E-8 has been reported to achieve high DON detoxification efficiencies (>90%) under optimal culture conditions [6,7]. Similarly, recent studies on *Y. tibetensis* have shown that its purified DON-detoxifying enzyme (YoDDH) can detoxify approximately 73% of DON (100 μM) within 3 h *in vitro* [4]. Consistent with these reports, our purified YTDepA achieved a comparable *in vitro* detoxification rate of 79.46% after 48 h (Figure 3B). However, the engineered yeast strain *Sc*-PQQ-Dep achieved a 13.98% detoxification rate *in vivo* over 72 h. Based on our experimental results, this efficiency gap is likely caused by specific limiting factors within the engineered yeast.

The two-step epimerization strategy targeting the C3 position of DON is currently recognized as one of the most effective enzymatic detoxification routes [6,7]. Similar to the well-documented DepA/DepB system isolated from *D. mutans* 17-2-E-8, our YTDepA oxidizes DON to 3-keto-DON, which is subsequently reduced to 3-epi-DON by YTDepB. Stereochemical inversion at the C3 hydroxyl group drastically diminishes the toxin’s binding affinity to ribosomes, rendering 3-epi-DON effectively non-toxic to mammals and plants. However, the *in vivo* realization of this entire pathway in a eukaryotic chassis is inherently complex.

A major limiting factor identified in our system is the low expression of *PqqD*, which likely restricts the intracellular synthesis of PQQ, directly limiting the catalytic capacity of YTDepA, the first and rate-influencing step. PQQ biosynthesis, natively found in a restricted group of Gram-negative bacteria (such as *K. pneumoniae*), relies on the highly conserved pqq operon. Within this cluster, PqqD acts as an indispensable chaperone that interacts with PqqC to finalize crucial ring closure and oxidation steps [28]. As observed in our qRT-PCR analysis (Figure 6A), the transcriptional expression of *PqqD* was disproportionately lower than that of other *Pqq* genes. Because PqqD is an indispensable chaperone required for critical biosynthetic steps [15,28], this transcriptional deficiency likely creates a severe metabolic bottleneck, limiting the intracellular pool of available free cofactor.

Another crucial factor limiting the current *in vivo* detoxification efficiency is the spatial mismatch between the detoxification enzymes and the cofactor supply. In native bacterial hosts, PQQ-dependent dehydrogenases typically function within the periplasmic space [29]. To mimic this natural scenario for practical grain treatment, our system utilized the α-factor targeting sequence to direct YTDepA and YTDepB through the endoplasmic reticulum (ER) for extracellular secretion. However, the multi-gene PQQ biosynthesis cluster was expressed within the yeast cytosol [29]. Consequently, the availability of intracellularly synthesized PQQ to the extracellularly secreted YTDepA is likely restricted by membrane transport or diffusion limitations, creating a significant spatial bottleneck [30]. As astutely suggested by recent theoretical insights, an advanced optimization strategy would involve targeting the PQQ synthetic pathway itself directly to the ER. The yeast ER is rich in S-adenosylmethionine (SAM) [31], which is an essential co-substrate for the radical SAM enzyme PqqE involved in PQQ biosynthesis [32]. Co-localizing the cofactor synthesis with the enzyme secretory pathway in the ER would fundamentally resolve this spatial mismatch and significantly enhance the detoxification efficiency of our yeast cell factory in future industrial applications. Additional host-specific challenges, such as those related to heterologous protein folding, post-translational modification, or substrate accessibility, may also be invovled.

In addition to the PQQ bottleneck for YTDepA, the availability of the redox cofactor NADPH poses another critical constraint for the downstream reductase, YTDepB. In this study, the *in vivo* decontamination assay was conducted in the standard YPD medium. Under such high-glucose conditions, *S. cerevisiae* predominantly undergoes fermentative metabolism due to the Crabtree effect [33]. Glycolysis and fermentation primarily generate and cycle NADH/NAD^+^, whereas the cytosolic NADPH pool—mainly supplied by the pentose phosphate pathway—may be insufficient to meet the high demand of the heterologous NADPH-dependent YTDepB. This cofactor mismatch likely restricts the second step of the epimerization process. Future optimization strategies must deeply consider the carbon source [34]; for instance, substituting glucose with non-fermentable carbon sources (such as glycerol) to shift the metabolic flux toward respiration, or genetically engineering the host’s endogenous NADPH regeneration cycles (e.g., overexpressing *ZWF1* or *ALD6*) [35], could significantly alleviate this metabolic bottleneck.

Therefore, this first-generation integrated strain not only validates the engineering strategy but also serves as a diagnostic platform. It clearly identifies priority targets for future optimization, such as enhancing PQQ biosynthesis pathway, engineering host metabolism, or evolving enzyme variants more compatible with the yeast intracellular environment, to unlock the full potential of this self-contained biocatalytic system for mycotoxin remediation. Future engineering efforts may also benefit from coupling this system with specific cellular transporters. For instance, co-expressing DON-specific efflux pumps or leveraging subcellular localization strategies (e.g., targeting the YTDepA/B enzymes to acidic organelles like the yeast vacuole) could circumvent current physiological constraints and dramatically enhance whole-cell catalytic efficiency [36].

## 5. Conclusions

In summary, we have successfully constructed a novel, self-contained *S. cerevisiae* platform that combines the entire DON epimerization pathway with its requisite cofactor synthesis. While the current detoxification efficiency highlights areas for improvement, this work establishes a foundational chassis and provides clear engineering directives. Future work will focus on boosting PQQ production through PqqD optimization, engineering yeast for improved enzyme performance (e.g., via secretion or cytosolic conditioning), and scaling up the fermentation process. This integrated biological strategy presents a promising and scalable alternative to conventional methods for mycotoxin mitigation in the food chain.

## Figures and Tables

**Table 1:** Key strains and plasmids used in this study.

Designation	Description	Key Features/Purpose
*Sc*-PQQ	*S. cerevisiae* CEN.PK2 with integrated PQQ gene cluster (pqqABCDEF)	PQQ-producing yeast chassis LEU2, TRP1 pTEF1, pPGK1
*Sc*-PQQ-YTDep	*Sc*-PQQ with integrated YTDepA and YTDepB expression cassettes	Final engineered strain for DON detoxification URA3, LEU2, TRP1 pTEF1, pPGK1
pYDepA	pETDuet-YTDepA	For recombinant YTDepA expression in *E. coli* Amp pT7
pYDepB	pET24a-YTDepB	For recombinant YTDepB expression in *E. coli* Amp pT7
pUC57-PQQ-Cluster	pUC57-based vectors for PQQ cluster integration (Figure S3)	For chromosomal integration of PQQ genes in yeast LEU2, TRP1 pTEF1, pPGK1

## Data Availability

The raw data supporting the conclusions of this article will be made available by the authors on request.

## Supplementary Materials

The following supporting information can be downloaded at https://www.mdpi.com/article/10.3390/biology15080654/s1. Figure S1: Chemical structure of DON; Figure S2: Diagram of the DON redox pathway; Figure S3: Plasmid construction map. Panels A–F illustrate the integration process of the DON-detoxifying enzyme genes, while panels G–K illustrate the integration process of the PQQ genes. Figure S4: Western blot analysis of target proteins YTDepA. Lane 1: CEN.PK2 control; 2: YTDepA protein expression sample 1; 3: YTDepA protein expression sample 2; M1: Protein Marker. Figure S5: Western blot analysis of target proteins YTDepB. Lane 4: CEN.PK2 control; 5: YTDepB protein expression sample 1; 6: YTDepB protein expression sample 2; M2: Protein Marker. Table S1: Additional plasmids used in this study. Table S2: DNA sequence of primer.
