# Supplementary material for: A Novel Genetic Engineering Approach for DON Detoxification Using a Yeast-Based Multi-Enzyme System
Source: Biology (Basel). 2026 Apr 21;15(8):654. doi: 10.3390/biology15080654 (PMC13112984; doi:10.3390/biology15080654)
Supplement: Supplementary file 1 [file biology-15-00654-s001.zip › biology-4207969-supplementary.pdf]

Supplementary

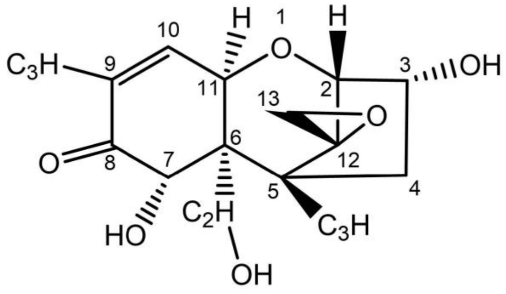

Figure S1. Chemical structure of DON

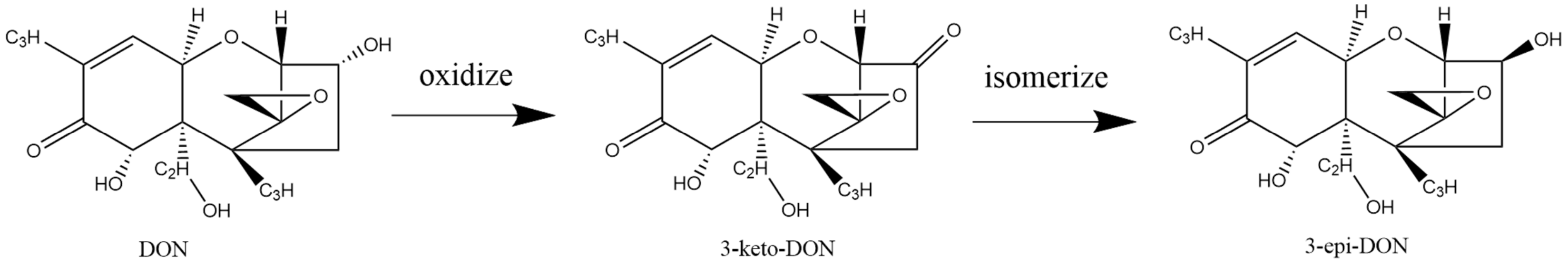

Figure S2. Diagram of the DON redox pathway

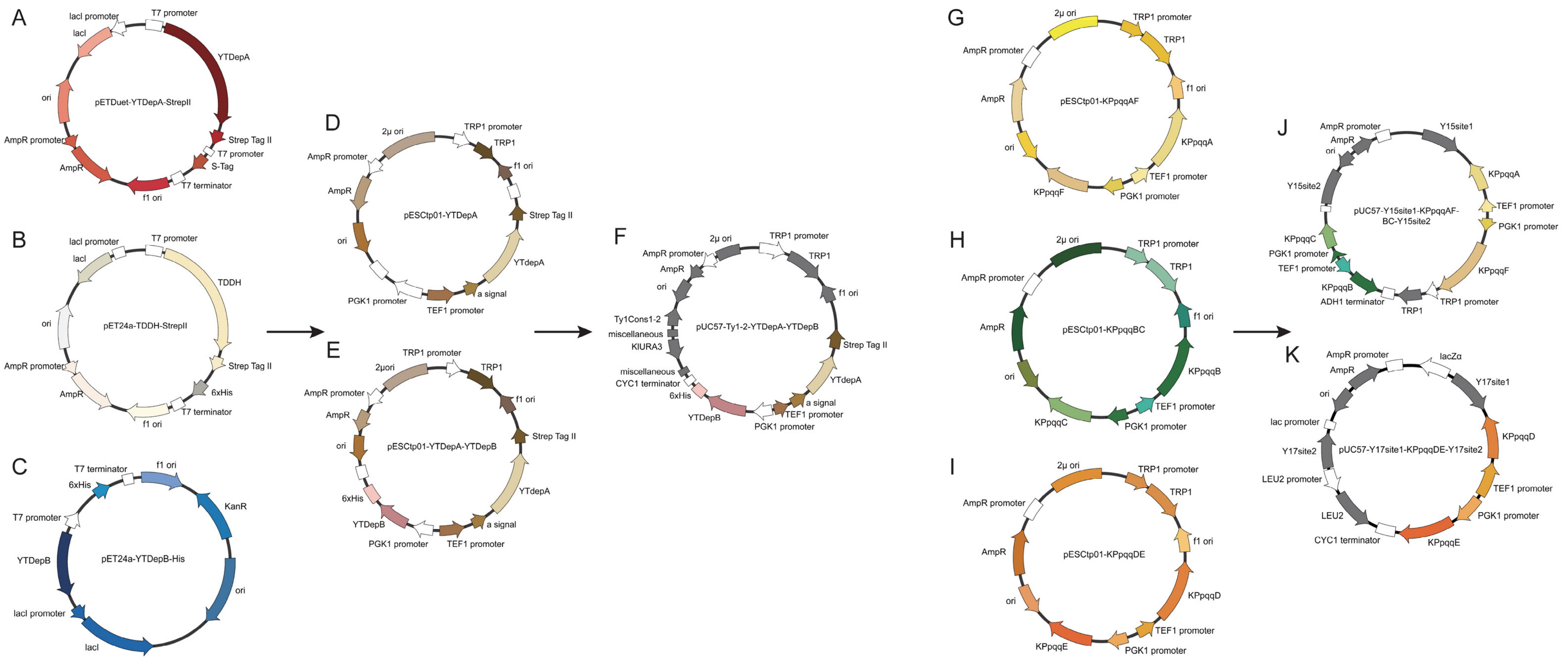

Figure S3 Plasmid construction map.Panels A–F illustrate the integration process of the DON-detoxifying enzyme genes, while panels G–K illustrate the integration process of the PQQ genes.

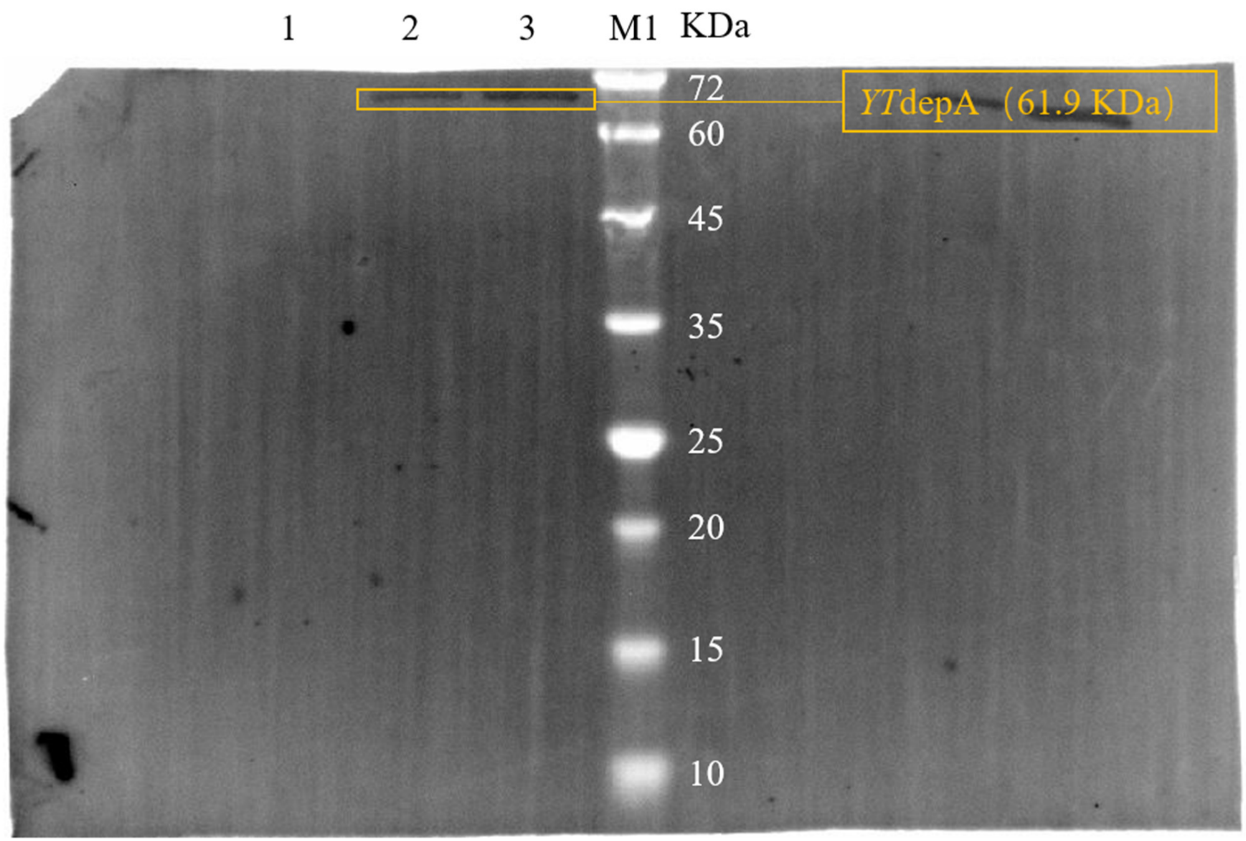

Figure S4 Western blot analysis of target proteins YTDepA. Lane 1: CEN.PK2 control; 2: YTDepA protein expression sample 1; 3: YTDepA protein expression sample 2；M1：Protein Marker.

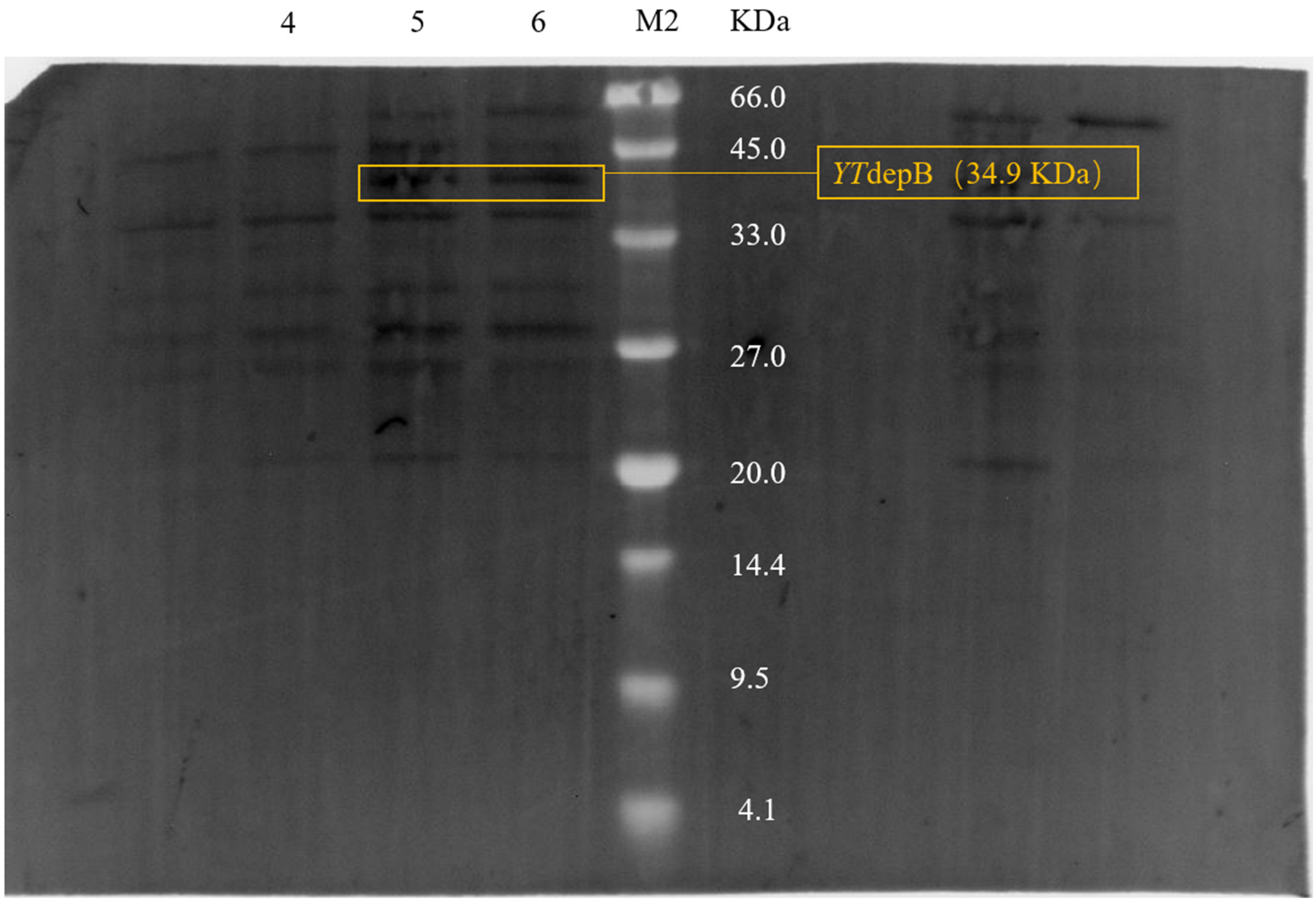

Figure S5 Western blot analysis of target proteins YTDepB. Lane 4: CEN.PK2 control; 5: YTDepB protein expression sample 1; 6: YTDepB protein expression sample 2；M2：Protein Marker.

Table S1 Additional plasmids used in this study.

Plasmids	Types of promoters	Resistance markers
pETDuet-YTDepA-StrepII	T7	Amp
pET24a-TDDH-StrepII	T7	Amp
pET24a-YTDepB-His	T7	Kan
pESCtp01-YTDepA	TEF1	Amp
pESCtp01-YTDepA-YTDepB	TEF1，PGK1	Amp
pUC57-Ty1-2-YTDepA-YTDepB	TEF1，PGK1	URA3
pESCtp01-KPpqqAF	TEF1，PGK1	Amp
pESCtp01-KPpqqBC	TEF1，PGK1	Amp
pESCtp01-KPpqqDE	TEF1，PGK1	Amp
pUC57-Y15site1-KPpqqAF-BC-Y15site2	TEF1，PGK1	TRP1
pUC57-Y17site1-KPpqqDE-Y17site2	TEF1，PGK1	LEU

Table S2 DNA sequence of primer.

Primers	Sequence
YTDepA for ETDuet-F	ACTTTAAGAAGGAGATATACATGCAGCACGCCGATGGGGCCGC
YTDepA for ETDuet-R	TTAAGCATTATGCGGCCGCATTATTTTTCAAACTGCGGATGTGACCATGCGCTCTTGGCTTCCGGCAGGGCGAAG
TDDH-F(Gib)	TTTAACTTTAAGAAGGAGATATACAATGCAGACCGCAATTAGCGATC
TDDH-R(Gib)	GGTGCTCGAGTGCGGCCGCATTATTTTTCAAACTGCGGATGTGACC
depB 24-F(Gib)	CTTTAAGAAGGAGATATACAATGGACGAAACCACCTCGCACC
depB 24-R(Gib)	AAGCTTGTCGACGGAGCTCGTTAGTGGTGGTGGTGGTGGTGGCG
KPpqqA-F	CAATCTAATCTAAGTTTTAATTACAAAGATGTGGAAGAAGCCAGCTTTC
KPpqqA-R	CGAAGAATTGTTAATTAAGAGCTTTATCTGTTAGAGATGTACAGGGTG
KPpqqF-F	CTACTTTTTACAACAAATATAAAACAGATGACTTTGGCTACTAGAAC
KPpqqF-R	ATCTTAGCTAGCCGCGGTACTCAGTCACCAGTAGTGAAC

KPpqqB-F	CAATCTAATCTAAGTTTTAATTACAAAGATGTTCATCAAGGTTTTGGG
KPpqqB-R	CGAAGAATTGTTAATTAAGAGCTTCAGCAAGCAGTATCTTGC
KPpqqC-F	CTACTTTTTACAACAAATATAAAACAGATGTTGATCACCGATACTTTG
KPpqqC-R	ATCTTAGCTAGCCGCGGTACTCAAACCAATCTAGTTGTATGC
KPpqqD-F	CAATCTAATCTAAGTTTTAATTACAAAGATGCAAAAGACCTCTATCG
KPpqqD-R	CGAAGAATTGTTAATTAAGAGCTTTACTCTGGTTCTCTACAAG
KPpqqE-F	CTACTTTTTACAACAAATATAAAACAGATGTCCCAATCTAAGCCAAC
KPpqqE-R	ATCTTAGCTAGCCGCGGTACTTACAAGTCTCTGGTTTGGTAG
Y15site1-F	TTGTAAAACGACGGCCAGTGGCCAGGCGCCTTTATATCATATAATTAAG
Y15site1-R	GCTCGGATCCCTACAAAATGAATCTACATTTCATTTTATTACGTC
Y15site1 insert1-F	CATTTTGTAGGGATCCGAGCGACCTCATGCTATACC
Y15site1 insert1-R	TTGGGCCCGGATCCCTTCGAGCGTCCCAAAACC
TRP-F	TCGAAGGGATCCGGGCCCAACGACATTACTATATATATAATATAGGAAGC
TRP-R	TAGATGCATTCGCGAGGTACGGGCCCGTATTTCACACCGCATAGGC
Y15site1 insert2-F1	AATGCATCTAGATATCGGATCGAGCTCGAGCGACCTCATGCTATACC
Y15site1 insert2-R	CATTGAGCTCCTTCGAGCGTCCCAAAACC
Y15site2-F(SacI)	ACGCTCGAAGGAGCTCAATGGAAGGTCGGGATGAGC
Y15site2-R	TTGCATGCAGGCCTCTGCAGATAAAGCAGCCGCTACCAAAC
Y17site1-F	TTGTAAAACGACGGCCAGTGGTGCACAAAGGCCATAATAT
Y17site1-R	GTCGCTCAGGCCTGAGTTATGGTTGCACAGTTACC
Y17site1 insert1-F	ATAACTCAGGCCTGAGCGACCTCATGCTATACCTG
Y17site1 insert1-R1	GTGGGGCCCAGGCCTCTTCGAGCGTCCCAAAACCTTC
LEU-F	CGAAGAGGCCTGGGCCCCACGTTGAGCCATTAGTATC
LEU-R	TAGATGCATTCGCGAGGTACGGGCCCCAAGAAATATCTTGACCGCAG
Y17site2-F	ATGCATCTAGATATCGGATCCTGAAACGCAAGGATTGATAATG
Y17site2-R	GATTACGCCAAGCTTGCATGAAAGCTGGCTCCCCTTAGAC
alpha YTdepA-F	TCTAATCTAAGTTTTAATTACAAAGAAAACAATGAGATTTCCTTCAATTTTTACTG

alpha YTdepA-R	CAGCATGTTGCCTTTTCTCGAGAGATACCC
depA-ESC-F	CGAGAAAAGGCAACATGCTGATGGTGCTG
depA-ESC-R	CGAAGAATTGTTAATTAAGAGCTTCACTTTTCGAATTGTGGATGTG
alpha YTdepB-F	ACTTTTTACAACAAATATAAAACAGAAAACAATGAGATTTCCTTCAATTTTTACTG
alpha YTdepB-R	TAGTTTCGTCCCTTTTCTCGAGAGATACCC
depB-ESC-F	CGAGAAAAGGGACGAAACTACCTCTCACTTG
depB-ESC-R	ATCTTAGCTAGCCGCGGTACTTAGTGATGGTGATGATGATGTC
In Ty-F	TCATAAATCATAAGAAATTCGCGGACCAAACCTCTGGCGAAG
In Ty-R	TCTGCAGTCGACGGGCCCGGCTTAGCTAGCCGCGGTACTTAG
β-actin-F	ACTTTCAACGTTCCAGCCTTC
β-actin-R	CGTAAATTGGAACGACGACGTGAGTA
RT-YTDepA-F	ACGCCAACGATGTTATTCA
RT-YTDepA-R	TCCAAAGCAACAACGAAAT
RT-YTDepB-F	CCTGCTGTTACTTCTGTTATT
RT-YTDepB-R	TCAGACATTTGTGGCATAG
RT-pqqB-F	TGCTCCAAGAACTCAATCC
RT-pqqB-R	GGTCAAGATAATACCACCAATA
RT-pqqC-F	ATTGGGTGAAGCTGTTGGT
RT-pqqC-R	AGACTGATGGATTTGTGGG
RT-pqqF-F	TTCTTTGACTCCACCACCA
RT-pqqF-R	GCAACCCATTGAGCATCTT
RT-pqqD-F	ATCCAGAAGGTATGGCTAA
RT-pqqD-R	ACCACCAGCTTCAGGAAAT
RT-pqqE-F	TTGCCAGACAGGACAAAGA
RT-pqqE-R	TTCAGTCAAACCAATACCG
